# The Annotation of Zebrafish Enhancer Trap Lines Generated with *PB* Transposon

**DOI:** 10.3390/cimb44060178

**Published:** 2022-06-02

**Authors:** Wenzhu Jia, Zhongxia Guan, Shasha Shi, Kuilin Xiang, Peihong Chen, Fen Tan, Numan Ullah, Mohamed Diaby, Mengke Guo, Chengyi Song, Bo Gao

**Affiliations:** College of Animal Science and Technology, Yangzhou University, Yangzhou 225009, China; jiawenzhu0215@outlook.com (W.J.); gzx18852728795@outlook.com (Z.G.); shashashi221@outlook.com (S.S.); 181903217@yzu.edu.cn (K.X.); cmxd18736647@outlook.com (P.C.); zmlzka@outlook.com (F.T.); numanhashmi25@gmail.com (N.U.); agrical6@yahoo.fr (M.D.); mk2528069423@outlook.com (M.G.); cysong@yzu.edu.cn (C.S.)

**Keywords:** annotation, *PB*, enhancer-trap, zebrafish

## Abstract

An enhancer trap (ET) mediated by a transposon is an effective method for functional gene research. Here, an ET system based on a *PB* transposon that carries a mini Krt4 promoter (the *keratin4* minimal promoter from zebrafish) and the *green fluorescent protein* gene (*GFP*) has been used to produce zebrafish ET lines. One enhancer trap line with eye-specific expression GFP named EYE was used to identify the trapped enhancers and genes. Firstly, GFP showed a temporal and spatial expression pattern with whole-embryo expression at 6, 12, and 24 hpf stages and eye-specific expression from 2 to 7 dpf. Then, the genome insertion sites were detected by splinkerette PCR (spPCR). The Krt4-*GFP* was inserted into the fourth intron of the gene *itgav* (*integrin*, *alpha V*) in chromosome 9 of the zebrafish genome, with the *GFP* direction the same as that of the *itgav* gene. By the alignment of homologous gene sequences in different species, three predicted endogenous enhancers were obtained. The trapped endogenous gene *itgav,* whose overexpression is related to hepatocellular carcinoma, showed a similar expression pattern as *GFP* detected by in situ hybridization, which suggested that *GFP* and *itgav* were possibly regulated by the same enhancers. In short, the zebrafish enhancer trap lines generated by the *PB* transposon-mediated enhancer trap technology in this study were valuable resources as visual markers to study the regulators and genes. This work provides an efficient method to identify and isolate tissue-specific enhancer sequences.

## 1. Introduction

Since the completion of the human gene map, humans have entered the post-genome era, and it has become a hot research direction to explore the functions of new genes and the new functions of known genes. At present, the commonly used research methods include gene bioinformatics analysis, gene spatiotemporal expression profile analysis, gene function prediction and experimental verification [1]. A gene trap is an important method used to find, identify and study a large number of unknown and known functional genes. It mainly includes enhancer trapping, promoter trapping and polyA trapping. Among them, an enhancer trap (ET) is a technique used to determine whether a DNA sequence contains enhancer functions, and it is an effective method to study and annotate the characteristics of enhancer-controlled gene spatiotemporal expression patterns in cells [2].

ET constructs usually consist of a reporter gene regulated by a weak promoter. When such a vector is integrated into the host genome, the promoter can be enhanced by the enhancers near the insertion site to drive the expression of the reporter [3]. The lentiviral vector system has become a commonly used vector for genetic modification due to its large gene fragments, high integration efficiency and persistent expression [4], but its biosafety has always been controversial. In contrast, the side effects of transposon on the host are far less than that of lentiviral vectors. Although the integration efficiency is not as good as that of lentiviral vectors, the transposition activity is extremely impressive. Enhancer trap mediated by transposon is a good application. *SB*, *PB* and *Tol2* transposons are currently the most commonly used in model animals [5,6,7,8]. Their trap efficiencies were found to all be above 80% in zebrafish, and they could all be reproductive. In addition, a transposon-mediated enhancer trap can produce a large number of mutants, and a mutant library with a stable inheritance of mutant traits can be established. Balciunas [9] used an *SB* transposon to carry out enhancer trapping studies in zebrafish and obtained nine zebrafish strains with different tissue or organ-specific *GFP* expression patterns. The transposons make it easier to obtain the sequences flanking the insertion sites and furtherly annotate enhancers.

Here, we annotate one *PB* transposon-mediated enhancer trap zebrafish line EYE, an eye-specific-expressing GFP line, using spPCR, in situ hybridization (ISH) and comparative genomics, aiming to establish an effective method in zebrafish to characterize enhancers in the genome, find novel patterns of gene expression and mutagenesis, and obtain other regulators.

## 2. Materials and Methods

### 2.1. GFP Detection of Enhancer-Trapped Zebrafish Lines

**Figure 1:**
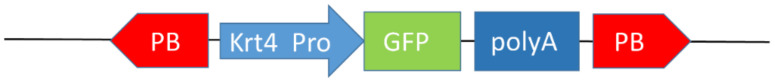
ET vector schematic. *PB*: two *PB* inverted terminal repeats (ITRs); Krt4 Pro: a minimal keratin4 promoter (the *keratin4* minimal promoter from zebrafish); *GFP*: *green fluorescent protein* ORF; polyA: rabbit β-globin polyA signal.

EYE zebrafish is a stable enhancer trapping strain prepared by our laboratory through *PB* transposon mediation. The enhancer trap vector contains a krt4 (*keratin4*) mini promoter and *GFP* expression cassette (Figure 1). The F2 generation embryos produced by F1 breeding were cultured in E3 medium in a 28 °C incubator. We observed and recorded the expression pattern of *GFP* embryos at 6 hpf, 12 hpf, 24 hpf, 2 dpf, 3 dpf, 4 dpf, 5 dpf, 6 dpf, and 7 dpf stages of embryos under a stereoscopic fluorescence microscope (M165FC, Lecia, Germany). After the fluorescence detection, the F2 generation zebrafish will continue to be reared for genome extraction.

### 2.2. PCR for Transgenes

To confirm the integration of an enhancer trap vector into a genome, a PCR was performed using two pairs of primers with the EYE and wild-type genome as templates, respectively. The primers were designed according to the *GFP* sequence and Krt4 promoter sequence (Table 1). If the EYE genome could use Krt4-F and *GFP*-R to amplify a 916 bp band (this band is the Krt4-*GFP* fragment in ET vector), and *GFP*-F and *GFP*-R to amplify a 720 bp band (this band is the *GFP* fragment in the ET vector), it meant that the reporter gene *GFP* was integrated into the genome. The PCR was performed under the following conditions: 1 cycle at 95 ℃ for 3 min; 34 cycles at 95 ℃ for 30 s, 60 ℃ for 30 s, 72 ℃ for 1 min; 1 cycle at 72 ℃ for 10 min. The products of PCR amplification were checked by agarose gel electrophoresis.

### 2.3. Splinkerette PCR for Genome Insertion Sites

The transposon insertion sites in the genome were detected by spPCR. The operation method for spPCR was performed as described in the literature [10]. The primers used for PCR amplification are shown in Table 1 and were designed as previously described [11]. Briefly, genomic DNA was isolated from F2 generation zebrafish using the TaKaRa Genome extraction kit (TaKaRa, Takara Biomedical Technology (Beijing) Co., Beijing, China) and digested with DpnI (TaKaRa, Takara Biomedical Technology (Beijing) Co., Beijing, China), followed by two rounds of PCR amplification. The specific steps are as follows. Genomic DNA were digested with DpnI, followed by two rounds of PCR amplification with primers specific for the transposon and splinkerette (Table 2). The first-round PCR was performed using the digested genomic DNA as the template with the primer pairs of SPLINK1/*PB*-SP1F under the following conditions: 1 cycle at 95 ℃ for 5 min; 35 cycles at 95 ℃ for 30 s, 60 ℃ for 30 s, 72 ℃ for 2 min; 1 cycle at 72 ℃ for 10 min. Then, the first-round PCR product (1 µL) was used as a template for the second-round PCR with primer pairs SPLINK2/*PB*-SP2F under the following conditions: 1 cycle at 95 ℃ for 5 min; 35 cycles at 95 ℃ for 30 s, 58 ℃ for 30 s, 72 ℃ for 1 min 30 s; 1 cycle at 72 ℃ for 10 min. The products of the second round of PCR amplification were purified and sequenced.

### 2.4. Enhancer and Endogenous Gene Annotation

The enhancer and endogenous gene were annotated in the 50 kb upstream and 50 kb downstream flanking genomic sequences of the transposon insertion site in zebrafish. The 100 kb genomic sequence of Zebrafish (GRCz11), the homologous sequences from Northern-pike (Eluc v4), Nile-tilapia (O_niloticus_UMD_NMBU), Mummichog (Fundulus_heteroclitus-3.0.2), Midas-cichlid (Midas_v5), Mexican tetra (Astyanax_mexicanus-2.0), and Amazon-molly (Poecilia_formosa-5.1.2) and gene annotation data were obtained from Ensembl. The obtained homologous sequences from these seven species of fish were aligned using the VISTA browser (http://genome.lbl.gov/vista/mvista/submit.shtml) (accessed on 14 October 2021) to identify the highly conserved non-coding sequences, which are annotated as putative enhancers. 

### 2.5. Whole-Mount In Situ Hybridization

To identify the expression profile of the endogenous gene *itgav* near the insertion site, the whole-mount in situ hybridization (WISH) on zebrafish embryos was performed as previously described [12]. Antisense RNA probes for target genes were synthesized according to the DIG RNA Labeling Kit (Roche). Embryos required for different periods were collected and fixed with paraformaldehyde (PFA). After permeabilization and prehybridization, hybridization was performed by incubating embryos with antisense DIG-labeled RNA probes overnight at 70 ℃, then followed by labeling reaction and staining, and images were captured using the M165 FC fluorescent microscope (Leica, Solms, Germany).

## 3. Results

### 3.1. GFP Expression Patterns in Zebrafish Enhancer Trap Line

**Figure 2:**
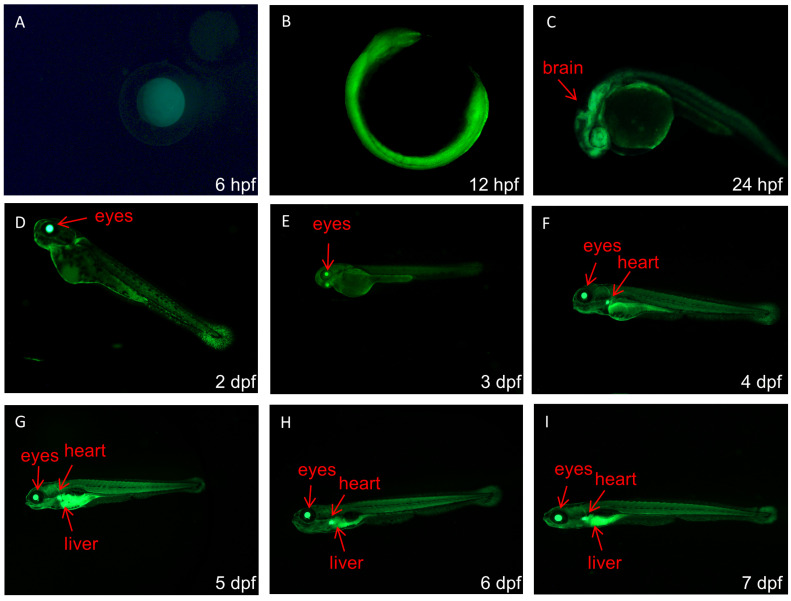
*GFP* expression patterns in the EYE line generated by the *PB*-mediated enhancer trap. (**A**–**I**) nine developmental stages. The arrows indicate GFP+ tissues.

The *GFP* fluorescence of EYE line F2 generation was observed at different developmental stages. As shown in Figure 2, from 6 to 12 hpf, the whole embryos showed enhanced fluorescence. At 24 hpf, green fluorescence in the brain was stronger than in other parts. From 2 to 7 dpf, the eyes showed strong green fluorescence; meanwhile, the fluorescence signal from the hearts gradually increased from 3 to 7 dpf. The fluorescence signal from the livers gradually increased from 4 to 7 dpf. These results showed the temporal and spatial expression characteristic of *GFP*, which was regulated by the trapped enhancers.

### 3.2. Insertion of Genome

**Figure 3:**
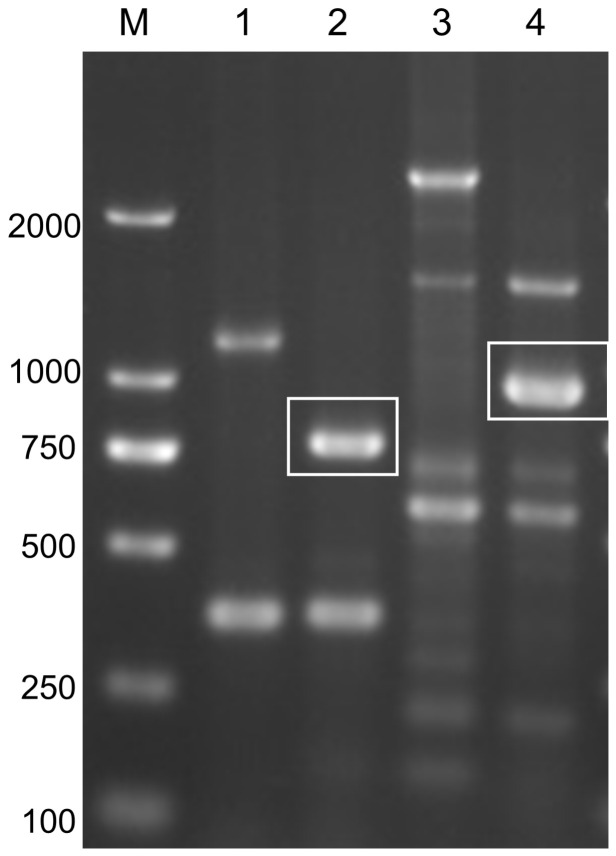
Electrophoresis image of PCR products in agarose gel. M: DL2000 bp marker; Line 1: the PCR product amplified from wild-type zebrafish genome by using *GFP*-F/R primers; Line 2: the PCR product amplified from EYE lines genome by using *GFP*-F/R primers; Line 3: the PCR product amplified from wild-type zebrafish genome by using Krt4-F and *GFP*-R primers; 4: the PCR product amplified from EYE lines genome by using Krt4-F and *GFP*-R primers. White boxes indicate the target bands (720 bp in Line 2, 916 bp in Line 4).

The insertion of the trap vector into the EYE lines genome was determined by PCR. The results showed two specific bands could be amplified from EYE, but not from wild type. The size of the two bands is in accordance with what is expected, 720 and 916 bp, respectively (Figure 3). The result suggested that the reporter gene *GFP* was integrated into the genome.

### 3.3. Enhancer Annotation

**Figure 4:**
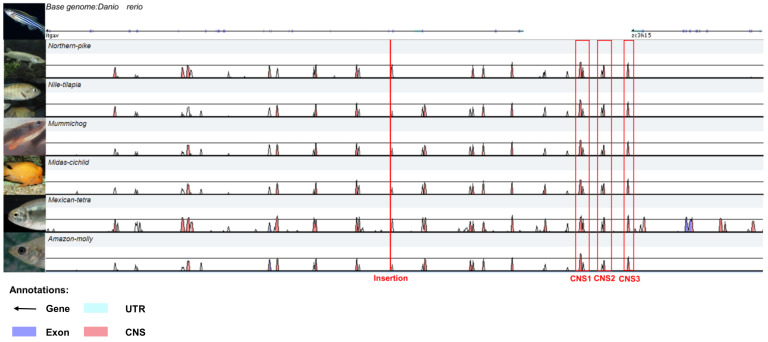
Enhancer distribution between 50 kb upstream and 50 kb downstream of the insertion site in the EYE lines genome. Genomic sequences from *Danio rerio*, *Northern-pike*, *Nile-tilapia*, *Mummichog*, *Midas-cichlid*, *Mexican tetra*, and *Amazon-molly* were analyzed in the VISTA browser. The red boxes represent three conserved non-coding sequences (CNS) that are predicted to be enhancers between *itgav* and *zc3h15 genes*. The red arrow indicates the insertion site of the ET vector. UTR: untranslated region.

**Figure 5:**
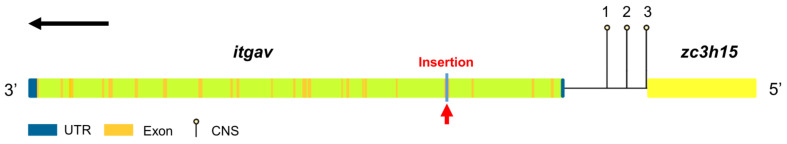
*Itgav* structural map. The red arrow indicates the insertion site of the ET vector. The black arrow indicates the orientation of the gene *itgav*. Numbers 1, 2, 3 indicate three conserved non-coding sequences (CNS) located upstream of *itgav* or downstream of *zc3h15*. UTR: untranslated region; CNS: conserved non-coding sequence.

The flanking DNA sequence of the *PB* transposon insertion site was aligned to the zebrafish genome database (GRCz10) from Ensembl. The result showed that the trap vector was inserted into the fourth intron of the endogenous gene *itgav* in chromosome 9. The insertion direction of the reporter gene is the same as that of the *itgav*. By aligning the 50 kb regions upstream and downstream of the insertion site across seven fish species, three conservative non-coding sequences (CNS) were found, which were in the upstream of *itgav* among seven species, Zebrafish, Northern-pike, Nile-tilapia, Mummichog, Midas-cichlid, Mexican tetra, and Amazon-molly (Figure 4). These three potential enhancers CNS1, CNS2, and CNS3 were distributed about 5–10 kb upstream of the *itgav* gene. The lengths of the three enhancers are 338, 92, and 132 bp. The structure of the *itgav* gene is shown in Figure 5. The *Itgav* (integrin, alpha V) gene is an endogenous gene on chromosome 9 of zebrafish, which is homologous to *human ITGAV* (integrin subunit alpha V). The whole size of *itgav* is 57,531 bp, with 30 exons and 29 introns.

### 3.4. Itgav Gene Expresstion Pattern

**Figure 6:**
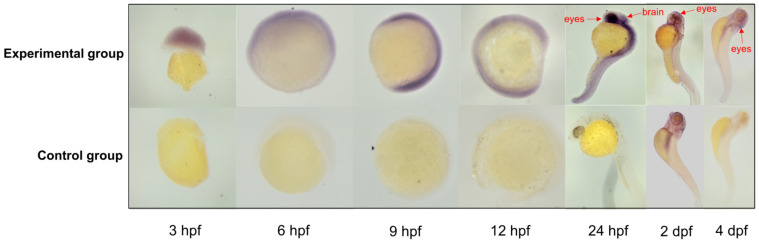
In situ hybridization for *itgav* transcripts at different stages of wide type zebrafish embryos. Red arrows indicate similar expression patterns to *GFP* in the EYE line. Experimental group: zebrafish embryos treated with *itgav* antisense RNA probes. Control group: zebrafish embryos without probe treatment.

The expression characteristic of *itgav* was detected by ISH on whole mount zebrafish embryos. The results showed that the *itgav* gene can be transcripted throughout the whole body in the early embryonic stages and specifically expressed in the eyes and forebody at 24 hpf, 2 dpf and 4 dpf. The staining of the eyes and brain were significantly stronger than in other parts of the embryos (Figure 6). The expression pattern of *itgav* was almost in consonance with that of *GFP*, which indicated that *GFP* can directly mark the expression patterns of the trapped endogenous genes.

## 4. Discussion

A selection of suitable transposons is important for a transposon-mediated enhancer trap. *PiggyBac* transposon was found in cabbage looper moth Trichophsiani. Its discovery enabled the application of transposon to achieve a breakthrough from lower organisms to mammals [13]. *PB* is an efficient tool for gene delivery and is, therefore, widely used in enhancer trap screens, given its biased nature towards transcriptional units, transcription start sites (TSSs), CpG islands, and open chromatin in general [14,15,16,17]. In our previous research, we showed that *PB*-mediated ET can produce more expression patterns of offspring and easily generate a mutant library in zebrafish [8]. Here, we annotated an ET zebrafish line mediated by *PB* and found the insertion site was inside an active expression gene in the genome, which was consistent with the *PB* preference for transcriptional units. We also found three CNSs upstream of the endogenous gene, which means the CNSs could be enhancers. This suggests *PB*-mediated ET was effective in trapping the regulatory regions in the genome.

In general, it is not difficult to generate transposon-mediated ET transgenics lines and characterize insertion sites in zebrafish. Actually, by a single cross of the founders in our research, more transgenic fish containing the expression of the reporter gene *GFP* can be provided. Those individuals with typical expression patterns were selected to establish lines. Here, the ET vector was inserted into the *itgav* gene of zebrafish chromosome 9. The expression pattern of *GFP* was basically the same as that of *itgav* in situ hybridization. This suggested *GFP* expression is driven by the nearby enhancers or regulatory regions and may reveal tissue-specific and cell-specific expression patterns of genes and enhancers. Collectively, *PB*-mediated ET makes it feasible to perform high-throughput enhancer screening.

Here, the ET construct can effectively detect enhancers in zebrafish genome. The marker gene was driven by a minimal promoter Krt4 derived from zebrafish and widely used in enhancer capture vectors [18,19]. However, promoters and enhancers have complex interaction effects, which are affected by transcription factors, folding factors, non-coding RNA, and histone modifications and other regulatory factors [20]. They are not a simple one-to-one correspondence but a many-to-many relationship. That means minimal promoter Krt4 cannot be working for all enhancers. If more enhancers need to be identified, multiple ET vectors with different mini promoters need to be used.

Taken together, a *PB*–transposon-combined enhancer trap provides an efficient approach to producing zebrafish mutant lines, which can be used as living markers to study gene expression and regulation.

## 5. Conclusions

In this work, we predicted three endogenous enhancers that regulate the expression of *itgav* genes by bioinformatics methods. The results showed that a *PB*–transposon-combined enhancer trap provides an efficient approach to producing zebrafish mutant lines, which can be used as living markers to study gene expression and regulation.

## Figures and Tables

**Table 1:** Primers for PCR.

Name	Sequence (5′–3′)
Krt4-F	GTGTGTGTGTGAGCAGTCAG
*GFP*-F	CACCATGGTGAGCAAGGGCG
*GFP*-R	TTGTACAGCTCGCCATGCCGA

**Table 2:** SP-PCR primers and linker sequences.

Name	Sequence (5′–3′)
SPLINK 1	CGAAGAGTAACCGTTGCTAGGAGAGACC
*PB*-SP1F	GACCTGCAGCCCAAAACTAA
SPLINK 2	GTGGCTGAATGAGACTGGTGTCGAC
*PB*-SP2F	ACCGATAAAACACATGCGTCA
SPLNK-GATC-TOP	GATCCCACTAGTGTCGACACCAGTCTCTAATTTTTTTTTTAAAAAAA
SPLNK-BOT	CGAAGAGTAACCGTTGCTAGGAGAGACCGTGGCTGAATGAGACTGGTGTCGACACTAGTGG

## Data Availability

Not applicable.

## References

[1] Du Y., Zuo Z. (2008). Advances in research methods for gene function. Chin. Bull. Life Sci..

[2] Liu C., Song G., Mao L., Long Y., Li Q., Cui Z. (2015). Generation of an Enhancer-Trapping Vector for Insertional Mutagenesis in Zebrafish. PLoS ONE.

[3] Bellen H., O’Kane C., Wilson C., Grossniklaus U., Pearson R.K., Gehring W.J. (1989). P-element-mediated enhancer detection: A versatile method to study development in Drosophila. Genes Dev..

[4] Naldini L., Trono D., Verma I. (2016). Lentiviral vectors, two decades later. Science.

[5] Zhong Y., Huang W., Du J., Wang Z., He J., Luo L. (2019). *Tol2* Improved -mediated enhancer trap identifies weakly expressed genes during liver and β cell development and regeneration in zebrafish. J. Biol. Chem..

[6] Parinov S., Kondrichin I., Korzh V., Emelyanov A. (2004). *Tol2* transposon-mediated enhancer trap to identify developmentally regulated zebrafish genes in vivo. Dev. Dyn. Off. Publ. Am. Assoc. Anat..

[7] Grabher C., Henrich T., Sasado T., Arenz A., Wittbrodt J., Furutani-Seiki M. (2003). Transposon-mediated enhancer trapping in medaka. Gene.

[8] Shen D., Xue S., Chan S., Sang Y., Wang S., Wang Y., Chen C., Gao B., Mueller F., Song C. (2018). Enhancer Trapping and Annotation in Zebrafish Mediated with *Sleeping Beauty*, *piggyBac* and *Tol2* Transposons. Genes.

[9] Balciunas D., Davidson A., Sivasubbu S., Hermanson S.B., Welle Z., Ekker S.C. (2008). Enhancer trapping in zebrafish using the *Sleeping Beauty* transposon. BMC Genom..

[10] Potter C., Luo L. (2010). Splinkerette PCR for mapping transposable elements in Drosophila. PLoS ONE.

[11] Sang Y., Shen D., Chen W., Chan S., Gu H., Gao B., Song C. (2018). Enhancer trapping nearby rps26 gene in zebrafish mediated by the *Tol2* transposon and it’s annotation. Sheng Wu Gong Cheng Xue Bao Chin. J. Biotechnol..

[12] Thisse C., Thisse B. (2008). High-resolution in situ hybridization to wholemount zebrafish embryos. Nat. Protoc..

[13] Fraser M.J., Smith G.E., Summers M. (1983). Acquisition of Host Cell DNA Sequences by Baculoviruses: Relationship Between Host DNA Insertions and FP Mutants of Autographa californica and Galleria mellonella Nuclear Polyhedrosis Viruses. J. Virol..

[14] Shima Y., Sugino K., Hempel C.M., Shima M., Taneja P., Bullis J.B., Nelson S.B. (2016). A Mammalian enhancer trap resource for discovering and manipulating neuronal cell types. Elife.

[15] Wang W., Lin C., Lu D., Ning Z., Cox T., Melvin D., Wang X., Bradley A., Liu P. (2008). Chromosomal transposition of *PiggyBac* in mouse embryonic stem cells. Proc. Natl. Acad. Sci. USA.

[16] Liang Q., Kong J., Stalker J., Bradley A. (2009). Chromosomal mobilization and reintegration of Sleeping Beauty and *PiggyBac* transposons. Genesis.

[17] Li M.A., Pettitt S.J., Eckert S., Ning Z., Rice S., Cadiñanos J., Yusa K., Conte N., Bradley A. (2013). The *piggyBac* Transposon Displays Local and Distant Reintegration Preferences and Can Cause Mutations at Noncanonical Integration Sites. Mol. Cell. Biol..

[18] Trinh L.A., Fraser S.E. (2013). Enhancer and gene traps for molecular imaging and genetic analysis in zebrafish. Dev. Growth Differ..

[19] Jhunjhunwala S., van Zelm M., Peak M.M., Cutchin S., Riblet R., Van Dongen J., Grosveld F.G., Knoch T.A., Murre C. (2008). The 3D Structure of the Immunoglobulin HeavyChain Locus: Implications for LongRange Genomic Interactions. Cell.

[20] Chatterjee S., Min L., Karuturi R.K.M., Lufkin T. (2010). The role of post-transcriptional RNA processing and plasmid vector sequences on transient transgene expression in zebrafish. Transgenic Res..

